# Congenital anomalies of the kidney and urinary tract: antenatal diagnosis, management and counselling of families

**DOI:** 10.1007/s00467-023-06137-z

**Published:** 2023-09-01

**Authors:** Emma Y. X. Walker, Paul Winyard, Matko Marlais

**Affiliations:** 1 Department of Paediatric Nephrology, Great Ormond Street Hospital for Children NHS Foundation Trust, London, WC1N 3JH UK; 2 UCL Great Ormond Street Institute for Child Health, London, UK

**Keywords:** Congenital anomaly of the kidney and urinary tract, CAKUT, Children, Ultrasound, Multidisciplinary team, Counselling

## Abstract

Congenital anomalies of the kidney and urinary tract are collectively one of the most commonly diagnosed antenatal conditions. Clinicians have several tools available to diagnose anomalies, including imaging, biomarkers, family history and genetic studies. In certain cases, antenatal interventions such as vesico-amniotic shunting may be considered to improve postnatal outcomes.

Congenital kidney anomalies detected antenatally can vary in clinical significance from almost no impact postnatally to significant morbidity and perinatal mortality. Prognosis broadly depends on kidney size, structure and amount of amniotic fluid, alongside genetics and family history, and progression on subsequent scans. It is important to counsel parents appropriately using a parent-focused and personalised approach. The use of a multidisciplinary team should always be considered.

## Introduction

Congenital anomalies of the kidney and urinary tract (CAKUT) are collectively one of the most diagnosed antenatal conditions, comprising around 20% of all congenital anomalies [1, 2]. CAKUT can range from mild unilateral hydronephrosis to dysplastic kidneys with multiple cysts and can have varying prognoses: some will have normal kidney function with no impact on a child’s day-to-day life, while others can lead to chronic kidney disease and kidney failure, requiring dialysis and transplant.

There are many tools available to use antenatally, including imaging, biomarkers, family history and genetic studies to confirm the diagnosis and likely prognosis as accurately as possible. This information then needs to be shared with the parents in a meaningful and accessible way, even when the content is distressing for parents to hear. A multidisciplinary team approach often helps here.

This article reviews antenatal aspects of CAKUT, highlighting the main sources of information for clinicians antenatally and discussing the process of antenatal prediction and counselling for parents.

## Embryology of the kidneys and urinary tract

To understand CAKUT malformations, it may be helpful to review the embryology of the kidneys and urinary tract and cellular mechanisms in kidney development. While this is outside the scope of this review, we would recommend reviewing Rosenblum et al.’s review alongside Davies and Bard’s book chapter [3, 4].

## Antenatal ultrasound

The kidney can be visualised on ultrasound from as early as 9 weeks, though it is not routinely sought on the 12-week scan. Assessment of the kidneys is part of the routine anomaly scan and is performed during the second trimester, generally between weeks 18 and 20 gestation but can be as late as 24 weeks in some countries. The information gained from the ultrasound is not limited to the anatomy of the kidneys and urinary tract; moreover, liquor volume, progression of changes and anomalies of other structures are important to consider too.

## Kidneys

### Agenesis and horseshoe kidneys

The first use of ultrasound is to identify the presence, or absence, of the kidneys. Isolated unilateral kidney agenesis occurs in 1 in 1000 to 2000 births, and approximately 1/3 of these will also have an associated abnormality [5, 6]. Horseshoe kidneys have an incidence of 1 in 400 to 500 births and commonly arise during development as a result of a fusion anomaly of the two kidneys. Both unilateral kidney agenesis and horseshoe kidneys can be benign; however, given that they can be associated with other CAKUT or extra-renal anomalies, there may be a risk of future complications [7, 8].

### Dilatation of the urinary tract

**Fig. 1:**
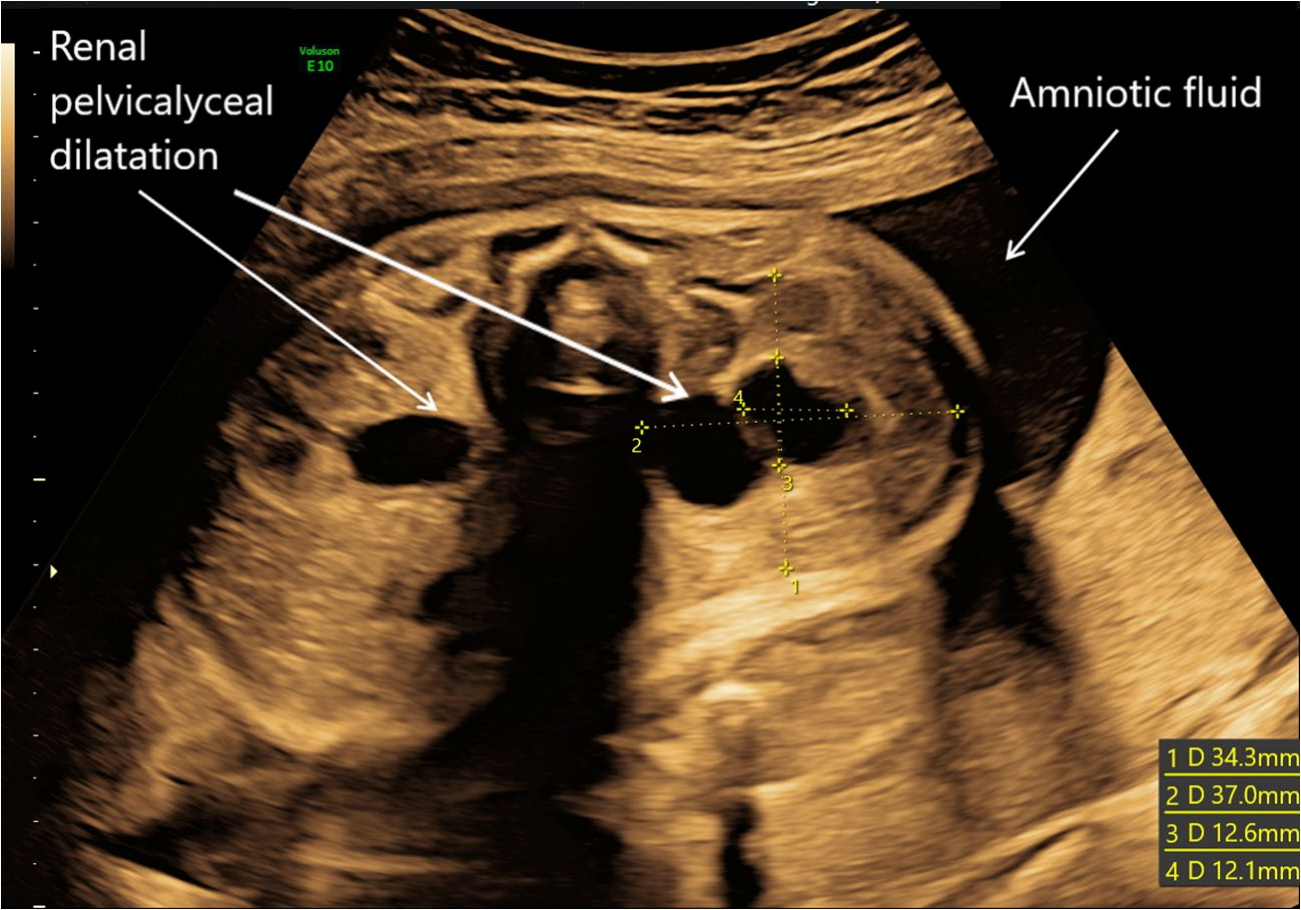
Antenatal ultrasound at 30/40 gestation showing bilateral renal pelvis and calyceal dilatation with normal amniotic fluid volume

Dilatation of the urinary tract is the most common abnormality detected antenatally and can vary from mild unilateral renal pelvis dilatation (RPD) to significant bilateral hydronephrosis and hydroureters. RPD is often measured antero-posteriorly in millimetres. The fetal kidney pelvis size increases almost linearly with gestational age with the 50th centile being at approximately 4 mm at 20 weeks gestation and 7 mm at term [9]. There has been an ongoing debate about the degree of urinary tract dilatation that is considered significant. The European Rare Kidney Disease Reference Network (ERKNet) stratifies prenatal risk into low and increased risk of lower urinary tract obstruction depending on RPD before and after 28 weeks gestation, with increased risk defined as RPD > 7 mm at < 28 weeks, > 10 mm at > 28 weeks, or any one of the following: dilation of peripheral calyces, abnormal parenchymal thickness or appearance, visibly dilated ureter, abnormal bladder and oligohydramnios [10, 11]. Figure 1 shows an example of bilateral RPD with normal amniotic fluid volume at 30 weeks gestation.

Renal pelvis dilatation can be subdivided into two groups: flow impairment in the upper and the lower parts of the urinary tract. Upper urinary tract dilatation is generally caused by obstructions or flow impairment at the pelvi-ureteric junction (PUJ) or vesico-ureteric junction (VUJ) but may also result from reflux and lower urinary tract obstructions.

**Fig. 2:**
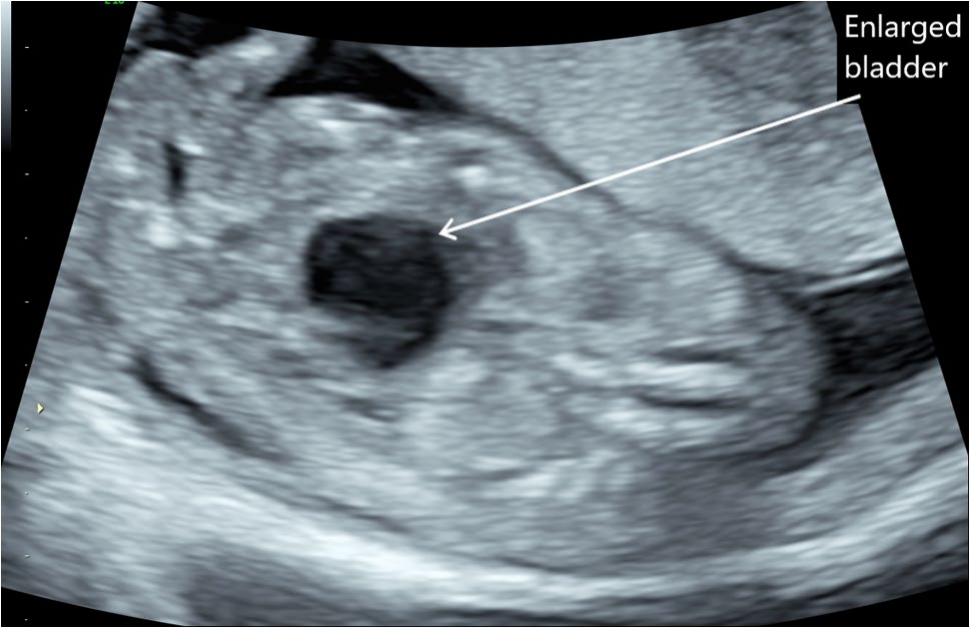
Antenatal ultrasound showing fetal megacystis (enlarged bladder) at 15/40 gestation

**Fig. 3:**
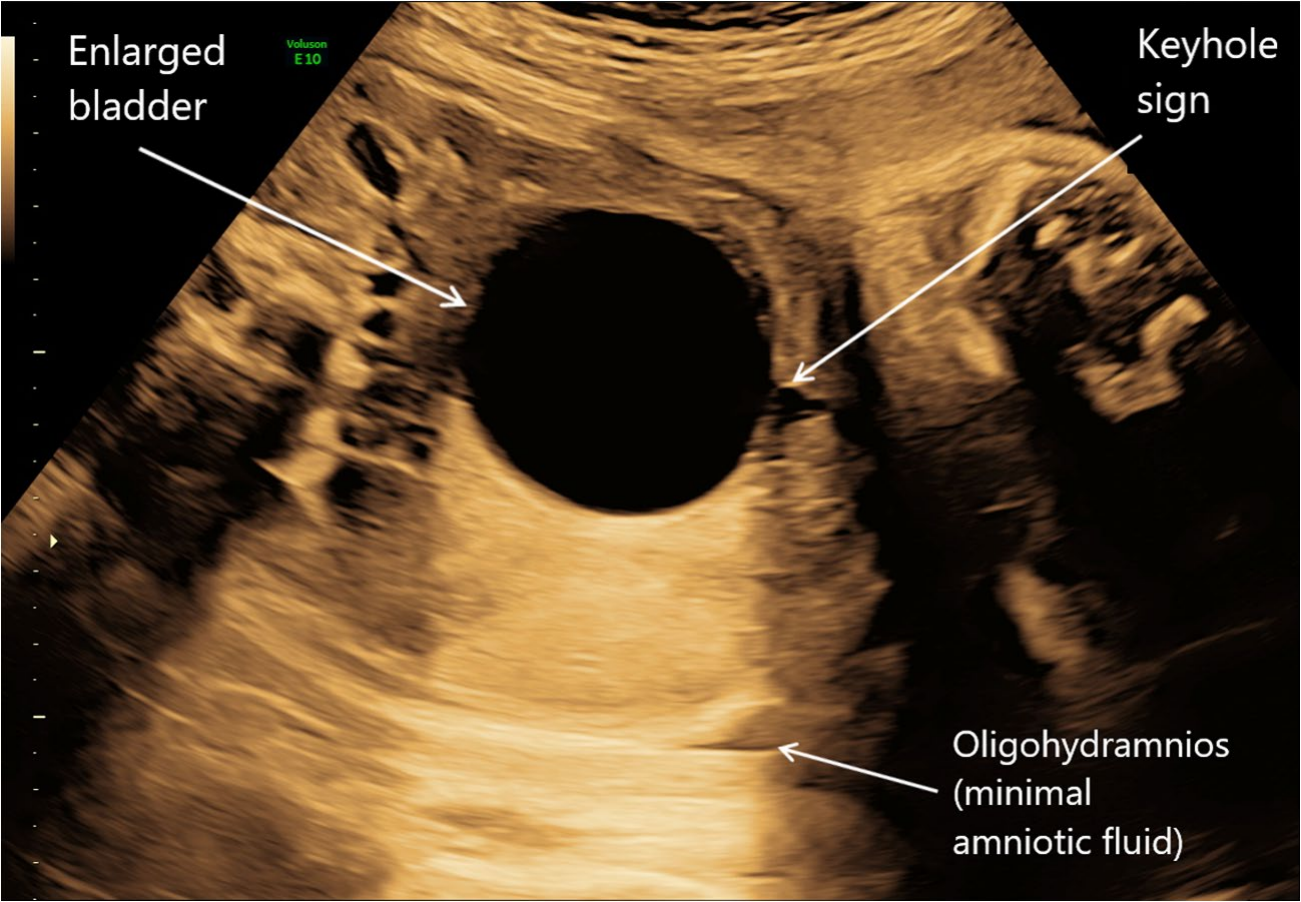
Antenatal ultrasound showing a distended bladder with ‘keyhole sign’ at 33/40 gestation with oligohydramnios. The infant was eventually confirmed as having posterior urethral valves postnatally

Lower urinary tract obstruction (LUTO) affects 2.2–3.3 per 10,000 births and is more common in boys than girls. The majority of these (62–64%) are caused by posterior urethral valves [12, 13]. However, other causes include urethral atresia/stenosis and prune belly syndrome. Early LUTO can be seen on antenatal ultrasound before 20 weeks gestation, as per Fig. 2 showing an example of megacystis at 15 weeks gestation. Features of LUTO on ultrasound are a distended, thick-walled bladder, with hydroureters and bilateral hydronephrosis; ultrasound can also be used to detect whether there are any associated kidney parenchymal anomalies which will guide staging of the LUTO [12]. The ‘keyhole’ sign is seen in certain cases of LUTO and is almost pathognomonic for posterior urethral values (see Fig. 3). There is significant morbidity and mortality associated with LUTO if the obstruction is significant enough. Morbidity includes pulmonary hypoplasia when associated with reduced amniotic fluid, and LUTO is one of the most common causes of kidney failure in children.

Those with antenatally detected urinary tract dilatation are at increased risk of urinary tract infections (UTIs) postnatally. If any dilatation of the urinary tract is identified antenatally, postnatal care will need to be adapted to account for this and ensure appropriate investigation and follow-up.

At present, the accepted practice in many countries continues to be the use of prophylactic antibiotics in the neonatal and infant periods where there is a significant unilateral or any bilateral renal pelvic dilatation, at least until postnatal imaging has further elucidated the situation. This is to prevent UTIs, kidney scarring and damage. However, the use of postnatal prophylactic antibiotics remains controversial. Antibiotics have been shown to modestly reduce UTIs in those who are pre-disposed to infections [14]. However, there remains an ongoing debate as to whether antibiotic use does reduce kidney scarring, and there is also the concern that unnecessary antibiotic use may lead to antibiotic resistance [15, 16]. Currently, the PREDICT trial is being conducted in over 40 European paediatric nephrology centres and is hoping to elucidate an answer to this debate through a prospective, multi-centre randomised control trial, the results of which would be interesting for any readers of this article.

### Dysplastic kidneys

A dysplastic kidney is a kidney that has abnormally developed in structure, and at times also in size, and the category in which some of the ciliopathies fall. In severe dysplasia, the kidneys have fewer glomeruli, collecting ducts and nephrons, and have increased stroma [13, 17]. Kidney dysplasia requires histological confirmation to definitively diagnose it; however, this is not often clinically or ethically appropriate. The 2022 ERKNet guidelines highlight the sonographic assessment that can be used as likely surrogate markers of kidney dysplasia to clinically diagnose kidney dysplasia [18]. Kidney dysplasia on antenatal ultrasound appears as increased echogenicity or ‘bright’ kidneys with poorly defined corticomedullary borders with or without cystic parenchymal changes [13]. Dysplastic kidneys can range from small/normal size for gestational age with no cysts, to over 9 cm in size with multiple large cysts; the latter of these may occur with multiple large cysts, echogenic stroma and no normal kidney tissue present, and this is termed *multicystic dysplastic kidney* [19]. While the diagnosis of a suspected multicystic dysplastic kidney can be made antenatally, caution must be taken, and appropriate postnatal imaging performed to confirm this and to ensure there is no communication between cysts as a severely hydronephrotic kidney (e.g. from PUJ obstruction) can masquerade as a suspected multicystic dysplastic kidney antenatally.

Unilateral dysplastic kidneys have an incidence of 1 in 3000–5000 births [19]. The differential diagnoses for dysplastic kidneys are wide-ranging, including aneuploidy, lower urinary tract obstruction, kidney cysts, diabetes syndrome, Bardet-Biedl syndrome and many other genetic syndromes; and it can also reflect a normal variant [20]. In view of the multiple aetiologies for dysplastic kidneys, the prognosis associated with the finding of a ‘bright’ kidney ranges vastly. Bilateral dysplastic kidneys occur less commonly and require more frequent monitoring as highlighted by the 2022 ERKNet consensus statement as they can be associated with an increased risk of decline of kidney function and thus CKD. However, there is variability with other factors such as UTIs, hypertension and proteinuria that contribute towards this decline, and so appropriate follow-up for these patients is required [18].

### Kidney cysts

Kidney cysts may also be detected later in pregnancy and these may be associated with ciliopathies. The subject of ciliopathies is outside the scope of this educational review, but clinical practice recommendations for the perinatal diagnosis and management of cystic kidney diseases were published in 2018 by Gimpel et al. and provide an extensive evidence-based resource to aid clinicians in their management of such cases [21].

### Amniotic pool

From approximately week 14 of gestation, the kidneys are the main contributor to amniotic fluid and, therefore, liquor volume on ultrasound. Thus, if there is an anomaly in the kidney, the liquor volume must always be assessed and monitored. Normal liquor volumes are generally reassuring and are estimated using the amniotic fluid index (AFI). This is a measurement of the amount of amniotic fluid seen and calculated on ultrasonography; an AFI of 5–25 cm is generally the accepted normal range. However, low volumes (oligohydramnios), or no liquor (anhydramnios) are concerning as they may affect lung development and can be associated with pulmonary hypoplasia and resultant poor postnatal respiratory outcome. Figure 3 shows an example of oligohydramnios in the context of LUTO. This, in turn, is associated with increased perinatal morbidity and mortality. Potter’s sequence, first described in 1946, is a rare and often fatal sequence, resulting from reduced/absent urine production (dysplastic kidneys) or severe LUTO which retains urine in the bladder (and has retrograde effects on the kidneys) leading to oligo/an-hydramnios and resultant pulmonary hypoplasia [22]. Oligo/an-hydramnios is the single most important predictor of postnatal kidney outcomes; therefore, its presence will have a substantial impact on the expected prognosis for a child after birth, and it plays a significant role in prenatal counselling [11].

### Other anomalies and syndromes

When an abnormality of the kidney is identified, it should always prompt the sonographer to review for any other anomalies. For example, kidney agenesis can be associated with other extra-renal anomalies in up to 30–31% of cases, such as Smith-Lemli-Optiz syndrome and renal-coloboma syndrome [6, 13]. Dysplastic kidneys can be seen in isolation, or as part of other syndromes, for example, VACTERL association, CHARGE syndrome and Bardet-Biedl syndrome (BBS) [13]. As an example, the finding of post-axial polydactyly in the context of large or echogenic kidneys should raise suspicion of BBS.

### Serial ultrasound scans

When an anomaly is detected, we believe that serial ultrasounds should be offered to monitor for any progression, e.g. dilatation of the urinary tract, or new formation of cysts, as well as monitoring amniotic fluid. The timing and frequency of serial ultrasounds will depend on the nature of the anomaly and its severity. This process is important not only in allowing clinicians to monitor over time, but it also allows parents time to come to terms with a diagnosis antenatally and make decisions about how to proceed, including those healthcare systems where termination of pregnancy (TOP) is legally permitted. Serial ultrasound scanning can also provide valuable information for obstetricians to plan aspects such as the timing and mode of delivery; as well as involving the neonatologists to have discussions regarding potential need for respiratory support, or, whether resuscitation is or is not offered at delivery. There is a variation between clinicians and healthcare systems in the frequency of repeated scans, which can in turn cause anxiety for parents. This is an area where evidence-based recommendations would be helpful.

### Limitations of ultrasonography

Ultrasonography is an important tool in identifying antenatal anomalies of the kidneys and guiding further discussions with parents regarding potential diagnoses. However, it is important to highlight that anomalies identified on ultrasound do not always correlate to pathological findings. Sun et al. performed a retrospective study in 1999 comparing prenatal ultrasound diagnoses with autopsy findings following termination of the pregnancy. The correlation was better for kidney anomalies over, for example, cardiac anomalies, with 63.6% complete agreement between ultrasound findings and autopsy findings. However, there were still 2 false-positive cases [23]. More recently, Scala et al., with a larger cohort, demonstrated an overall diagnostic accuracy of antenatal ultrasound to detect multicystic dysplastic kidneys of 91.3% [24]. Though there are still some that are incorrectly diagnosed, correlation between ultrasound findings and anatomical anomalies may be different with more modern ultrasound equipment.

Moreover, when ultrasounds do detect anomalies, they cannot accurately predict postnatal kidney function. As previously mentioned, amniotic fluid volume (or the lack of it) is the best indicator of postnatal kidney function from imaging, but even in the presence of oligo/an-hydramnios, there can still be a wide range of postnatal outcomes in terms of kidney function, especially when the lack of amniotic fluid is due to urinary tract obstruction which may be relieved postnatally. Furthermore, parents often focus on the more severe outcomes, which in term may cause more anxiety/depression than may be needed.

### Antenatal magnetic resonance imaging

While ultrasonography is the modality of choice for assessing the unborn baby, magnetic resonance imaging (MRI) is also used to evaluate some congenital anomalies of the fetus. Generally, fetal MRIs are performed to provide further information on the neurological system: the brain and the spine [25, 26]. MRI can, however, be considered as an adjunct to complement ultrasound images of the abdomen [27]. It has been used to assess both the kidney and lung parenchyma in further detail especially when ultrasound is inconclusive due to oligohydramnios and can also be used in those with suspected autosomal recessive polycystic kidney disease [28–30]. There is an ongoing debate regarding whether it should be performed routinely in all those with major structural anomalies detected [31]. Ultrasound will continue to be the imaging modality of choice, but fetal MRI may be considered a useful aide, especially when ultrasound is inconclusive. Fetal MRI may be particularly useful in cases with associated anomalies which may require surgical intervention, and antenatal planning of such cases may be beneficial.

## Biochemical markers

Biochemical markers are being researched and proposed as potential predictors of postnatal kidney function. β2-microglobulin from fetal serum has previously been used as an indirect measure of fetal GFR, with increased levels reflecting tubular damage. However, there is a lack of normative data and a lack of studies to support the routine use of this as a measure of fetal kidney function [32, 33]. One study found that in 34 surviving infants with bilateral obstructive uropathy, a fetal serum β2-microglobulin cutoff of 5 mg/L had a specificity of 100% for a serum creatinine ≤ 50 umol/L, but a sensitivity of 67% for a serum creatinine ≥ 50 umol/L at 6 months of life; however, the numbers in this study were small, limiting the conclusions [35]. Assessment of electrolytes in fetal urine (as a theoretical surrogate marker of fetal urine production and concentrating ability) has been studied to try and predict postnatal kidney prognosis. A systematic review of 23 studies including 572 women looking at the accuracy of fetal urine found that urinary calcium and sodium were the most useful analytes in fetal urine, but they concluded that no fetal urine analytes were sufficiently accurate to be of utility in clinical practice [34, 35].

Biomarkers for PUV have been analysed in fetal urine, and peptides have been identified that are specifically associated with PUV with early kidney failure [36]. The ANTENATAL multicentre study is currently being undertaken and is specifically evaluating fetal urinary omics traits to peptide markers for kidney disease stratification [37]. In the future, this may also contribute to decision-making and management of fetuses with PUV specifically.

## Genetics and family history

When performing the antenatal assessment, it is important to take a thorough family history of any kidney anomalies. For example, kidney agenesis has, since 1984, been identified as a potentially heritable condition [38]. More recently, studies have been performed to try to identify a genetic basis for kidney agenesis though it is not as clear as other diseases with very limited cases attributable to a monogenic cause [39].

An identified causative gene for congenital kidney anomalies is *HNF1β*. *HNF1β* encodes for a transcription factor which is important in both the kidney and pancreas organogenesis [40]. It is inherited in an autosomal dominant manner, with a high proportion of de novo variants. It can, however, present with multiple phenotypes. Prenatal phenotypes that have been documented are isolated bilateral hyperechogenic kidneys, bilateral multicystic dysplastic kidneys, unilateral kidney hypoplasia, and isolated upper urinary tract dilation, and the associated severity of the kidney disease is extremely variable [40]. It may also be part of a 17q12 microdeletion syndrome, with further associated problems such as learning difficulties.

A genetic diagnosis should be seen as an adjunct to ultrasonography, and a variant in an identified gene does not necessarily lead to a specific phenotype. It is important to be able to understand and interpret the genetic results, if available, as actioning a variant that has no, or low, risk of causing disease could also in itself have negative consequences. Next generation sequencing is emerging and enables sequencing of fetal exomes which can improve diagnostic yield. However, there must be the consideration of ethical issues around potential secondary findings and the counselling needed for this [41].

Additionally, in order to obtain these genetic samples, the mother must undergo either amniocentesis or chorionic villus sampling depending on the gestation at which the sample is obtained. It is well known that there are multiple risks associated with these procedures, including risk of congenital anomalies (i.e. talipes), spontaneous miscarriages and perinatal deaths [42]. A recent systematic review and meta-analysis suggest the risk of miscarriage is lower than quoted by healthcare professionals, but it is still a risk that needs to be considered nonetheless [43].

Free fetal DNA testing is an emerging non-invasive technique for prenatal diagnosis, mitigating many of the aforementioned risks, and it can be performed as early as 5–7 weeks gestation. It is, however, currently limited in what information can be gathered, notably sex chromosomes and aneuploidies such as trisomy 13, 18 and 21, as well as genetic disorders inherited from the father such as haemophilia. This is an active area of research, though, and there may be the potential for more in-depth fetal DNA analysis [44].

Thus, identifying genetic anomalies is not without ethical and medical implications. While genetic diagnoses can help families, they do not necessarily correlate with a specific phenotype as illustrated with *HNF1β* variants. Moreover, there may be an element of genetic guilt that parents may feel, passing on the mutated genes. Considering this, when performing any genetic investigation, open conversations should be had with parents and consent gained before pursuing a genetic diagnosis.

## Interventions

Interventions for congenital kidney anomalies vary from conservative management to fetal intervention and surgery. Fetal lower urinary tract obstruction has been managed with antenatal vesico-amniotic shunting (VAS) or serial amnioinfusion by many fetal medicine specialists over the last three decades, but the literature for these interventions remains controversial. A meta-analysis of studies performed in 2003 suggested a slight improvement in perinatal survival with the use of VAS, but the effect size was small [45]. Moreover, a further meta-analysis in 2017 concluded that there was a higher perinatal survival initially to 6 months, but that long-term data for 1-year survival and for kidney function remain uncertain [11, 46]. While some fetal medicine specialists continue to advocate the use of shunting, it remains difficult to provide a strong evidence base for its use. Improvements in survival may be related to improved postnatal lung function due to improved lung development with amniotic fluid present, but there is no convincing evidence that relieving the urinary tract obstruction improves postnatal kidney outcomes as it is likely that kidney dysplasia occurred early in fetal development. It is also important to note the risks associated with VAS, including risks of miscarriage along with other risks such as dislodgement of shunt and bladder rupture following shunt insertion [46].

ERKNet’s 2022 recommendations are for those in whom prenatal intervention is indicated following MDT discussion; they would recommend VAS placement, ideally prior to 27 weeks gestation [11]. However, a recent study compared VAS shunting at 3 different gestations: 12 + 5 to 16 + 0 weeks, 16 + 1 to 24 + 0 weeks, and 24 + 1 weeks and beyond. Their study demonstrated increased fetal survival if the VAS shunt was performed at a later gestation, but that earlier intervention resulted in a high chance for normal kidney function. This will be an area that will need to be studied further [47].

Fetal cystoscopy for the diagnosis and treatment of lower urinary tract obstruction with prenatal laser ablation of the PUV has also been studied. While some literature demonstrates there is some potential in improving postnatal outcome with fetal cystoscopy, a meta-analysis in 2011 concluded that there was very little evidence at present to support routine use of fetal cystoscopy, and further studies are required [48, 49]. Current NICE guidelines in the UK do not support the routine use of fetal cystoscopy unless there are special arrangements for consent for audit or research [50].

Given the wide variation of outcomes, the management of those with suspected CAKUT should be with a specialist multidisciplinary team.

## Counselling and prognosis

Thus far, we have reviewed that congenital kidney anomalies can be detected antenatally through ultrasound, or, through a risk based on family history and genetic analysis. We have also alluded to areas that could improve our ability to prognosticate, including other imaging such as MRI, ability to calculate a form of fetal glomerular filtration rate, or interventions such as fetal cystoscopy. The next stage is how to convey this information to the parents and how to address the psychosocial aspect of kidney anomalies and genetic diagnoses.

### Personalised approach

Needless to say, no parents or families are the same. With each consultation and counselling of parents of fetal anomalies, the practitioner needs to consider the individuality of the parent: ethnic and cultural beliefs, their age, the family history and passed lived experiences. An example is a small study in 2015 in which 12 pregnant Muslim Moroccan women were interviewed to review their preferences towards counselling for anomaly screening (note: this is not that anomalies were detected but the counselling of screening in the first place). Key aspects arose from the thematic analysis in this study and led to several recommendations: to have accurate and detailed information available, to ask the individual their views on life and be genuinely interested, to have practical knowledge of religious beliefs about the value of life, disabled life and termination, and to explore the role of religion in decision-making [51]*.* Their views are not limited to their belief or culture but could be applied to any individual. In summary, counselling should be personalised to facilitate parents making informed choices. Moreover, we should be aware of the impact such a diagnosis has on a family in terms of the wider family, the time commitment associated with increased hospital appointments and the potential lifetime of medicalisation for the unborn child. A personalised approach will improve the engagement that parents have with the understanding of their disease.

### Conveying uncertainty

It is important for us to be able to accurately interpret the information we have, though we often do not know the extent of the postnatal impact that the antenatal findings may have. As clinicians, uncertainty is often difficult to talk about especially when the outcome is bleak for fear of upsetting patients and their families [52]. Modern medicine has, however, shifted to shared and patient-informed decision-making. Therefore, it is important to convey uncertainty to patients, to enhance their knowledge in making decisions about investigations or treatment and to give patients their autonomy [53].

Another difficulty is how to portray population risk, which is how we, as practitioners, interpret results. For example, a disease will affect 1 in 200 (0.5%), but from the patient’s perspective, they either will or will not experience the outcome and may therefore consider it as more of a 50:50 situation. Discussing prognosis is important for patients to ‘hope for the best, while planning for the worst’ [52]. There is no best practice on how to convey uncertainty or prognosis, but it is an area of science and sociology that is expanding.

### Non-directive counselling

Non-directive counselling is where clinicians provide the relevant information and support to enable patients (or, in this case, parents) to make their own informed decisions, rather than clinicians giving advice about their beliefs on the best course of action and therefore directing the process. The entire concept of non-directive counselling has been questioned by clinicians and ethicists, as in practice, it is very difficult to be totally non-directive [54]. Parent groups have cited clearly that an appropriate handling of the process of antenatal counselling, including providing the best possible information in a timely way, can help to minimise the excess distress which may occur in an already very distressing situation [55].

### Anxiety and trauma

When a parent receives a diagnosis of a congenital anomaly, it undoubtedly leads to anxiety and distress. Marokaksi et al. performed a systematic review in 2016 to look at the evidence of the impact of non-genetic prenatal counselling on congenital anomalies. Only 3 of the 24 papers reviewed addressed parental anxiety, but each of these reported a significant decrease in anxiety following counselling [56]. Parents felt they should have counselling soon after the diagnosis is made to reduce the stress and anxiety associated with waiting.

In 2011, a team in Italy, studied whether a prenatal diagnosis of a congenital anomaly can be considered a traumatic experience as defined by the American Psychiatric Association since often the diagnosis (for example, based on abnormal ultrasound) is sudden and unexpected. A total of 145 out of 165 mothers, and 76 out of 91 fathers, experienced the communication of diagnosis as a traumatic event [57]. The study did not find a correlation between the potentially lethal conditions, or the ‘simple-to-fix’ anomalies, with the trauma experienced. From this, we can conclude the importance of effective communication and support for the family, regardless of the perceived severity of the anomaly and likely postnatal outcome.

There have also been studies reviewing how much information is recalled following counselling sessions. Most will recall the location and severity of the anomaly, but fewer can recall the prognosis or the cause. There have been variable outcomes from studies regarding the effectiveness of giving supplemental information in improving recall; however, information given in the form of written, visual and web-based resources was found to reduce parental anxiety [56].

### Multidisciplinary team approach

One way of improving the experience for parents is taking a multidisciplinary team approach. A team of fetal medicine obstetricians, alongside nephrologists, paediatric surgeons/urologists, neonatologists, geneticists and psychologists should be considered. Many antenatal counselling discussions in developed healthcare systems are initiated and led by fetal medicine specialists; while they are well-placed to start these discussions often based on antenatal ultrasound findings, they may not be able to answer all questions that families may have. For example, when counselling a family about a case of bilateral dysplastic kidneys with oligohydramnios, fetal medicine specialists are likely to know there is a significant chance of requiring dialysis in infancy or childhood, but they are unlikely to be able to give parents a full understanding of what the quality of life may look like for a child undergoing kidney replacement therapy in early childhood. Moreover, the value of being involved in antenatal counselling is twofold; not only does it help the patient and family, but also the clinicians, and it should be part of sub-specialist training for paediatric nephrologists [58]. In our experience, for cases where a poor perinatal prognosis is expected with a significant risk of mortality, counselling alongside involvement of palliative care teams can be helpful for families, preparing them for the likely postnatal events and focusing on keeping the newborn infant comfortable with conservative care, minimising interventions which may cause distress.

Of particular relevance for paediatric nephrologists who undertake antenatal counselling is the long-term impact of what is said and what is predicted. While clinicians make every effort to acknowledge the uncertainty in postnatal outcomes, this information is not always retained by parents in the midst of such a distressing situation, especially if they have just been told the news of a congenital anomaly. For this reason, separate counselling sessions may be beneficial to allow time for families to process information. It is important to note that if parents feel that they have been given inaccurate information or if clinicians are too absolute with their antenatal counselling, then families can lose trust with future clinicians if the antenatal predictions turn out to be incorrect. We therefore advocate for a non-directive counselling approach regarding decisions around TOP, and an approach that provides the best possible evidence-based information but acknowledges that there is always uncertainty, and some antenatal predictions will eventually turn out different to what is expected. This is especially important in a specialty like paediatric nephrology, where care of chronic kidney disease may proceed from infancy until early adulthood.

Finally, parents will also need support with future family planning for conditions where there is an inherited component. Again, an MDT approach should be taken with particular focus on involvement of the geneticists for genetic counselling.

## Conclusions

Kidney anomalies are the most commonly diagnosed antenatal congenital abnormality. They are usually detected on the 18–21-week fetal anomaly ultrasound scan but can be picked up earlier. It is important to assess not only the kidney structure and size but also the amniotic fluid level and any other congenital anomalies. Family history is an important part of history taking as part of the antenatal workup. Genetic testing should be considered and can be helpful to families; however, it is pertinent to remember that genetic variants may not correlate with specific phenotypes. Therefore, parents should be counselled prior, and results interpreted as an adjunct to other information available.

Congenital kidney anomalies detected antenatally can vary in clinical significance from almost no impact postnatally, to significant morbidity and perinatal mortality. Prognosis broadly depends on kidney size, structure and amniotic fluid, alongside genetics and family history, and progression (or not in some cases) on subsequent scans. It is important to counsel parents appropriately using a parent-focused and personalised approach. The use of the multidisciplinary team should always be considered antenatally.

In the future, further imaging modalities such as fetal abdominal MRI, may assist in providing a more accurate diagnosis antenatally and ultimately improve the information available for parents to make informed decisions. There is, however, likely to remain a significant amount of uncertainty and therefore the principles of non-directive antenatal counselling are paramount.

## Key summary points

- Ultrasonography (US) is first line in detecting any kidney abnormality and can be used to assess kidney size and structure and associated amniotic fluid level.
- Prognosis depends on US findings and their progression, alongside genetics and family history as adjuncts.
- Antenatal intervention can be considered for some congenital kidney anomalies, but when doing so, it should be with a large MDT.
- Non-directive counselling should be used to give parents relevant information and support them in making informed decisions.
- Counselling should be performed in a timely manner taking an MDT approach.

## Multiple choice questions 

Answers may be found following the references

1. By what week is fetal urine the major contributor to amniotic fluid?
  - 8 weeks
  - 14 weeks
  - 20 weeks
  - 24 weeks
2. What is the 50th centile for renal pelvis size at term?
  - 4 mm
  - 7 mm
  - 12 mm
  - 15 mm
3. Variants in *HNF1β* have been associated with which of the following prenatal phenotypes?
  - Bilateral multicystic dysplastic kidneys
  - Bilateral hyperechogenic kidneys
  - Unilateral kidney hypoplasia
  - Isolated upper urinary tract dilation
  - All of the above
4. True or false: prenatal diagnosis of a congenital anomaly can be considered a traumatic experience.
  - True
  - False

## Abbreviations

CAKUT: Congenital anomalies of the kidney and urinary tract
RPD: Renal pelvis dilatation
ERKNet: The European Rare Kidney Disease Reference Network
PUJ: Pelvi-ureteric junction
VUJ: Vesico-ureteric junction
LUTO: Lower urinary tract obstruction
UTI: Urinary tract infection
CKD: Chronic kidney disease
AFI: Amniotic fluid index
TOP: Termination of pregnancy
MRI: Magnetic resonance imaging
VAS: Vesico-amniotic shunting
NICE: National Institute for Health and Care Excellence
MDT: Multidisciplinary team
